# Enhancement of the anti-tumor activity of FGFR1 inhibition in squamous cell lung cancer by targeting downstream signaling involved in glucose metabolism

**DOI:** 10.18632/oncotarget.19279

**Published:** 2017-07-17

**Authors:** Claudia Fumarola, Daniele Cretella, Silvia La Monica, Mara A. Bonelli, Roberta Alfieri, Cristina Caffarra, Federico Quaini, Denise Madeddu, Angela Falco, Andrea Cavazzoni, Graziana Digiacomo, Giulia Mazzaschi, Valentina Vivo, Elisabetta Barocelli, Marcello Tiseo, Pier Giorgio Petronini, Andrea Ardizzoni

**Affiliations:** ^1^ Department of Medicine and Surgery, University of Parma, Parma, Italy; ^2^ Food and Drug Department, University of Parma, Parma, Italy; ^3^ Medical Oncology Unit, University Hospital of Parma, Parma, Italy; ^4^ Division of Medical Oncology, Sant'Orsola-Malpighi University Hospital, Bologna, Italy

**Keywords:** FGFR1, glucose metabolism, dovitinib, NVP-BGJ398, SQCLC

## Abstract

Fibroblast Growth Factor Receptor (FGFR) signaling is a complex pathway which controls several processes, including cell proliferation, survival, migration, and metabolism. FGFR1 signaling is frequently deregulated via amplification/over-expression in NSCLC of squamous histotype (SQCLC), however its inhibition has not been successfully translated in clinical setting. We determined whether targeting downstream signaling implicated in FGFR1 effects on glucose metabolism potentiates the anti-tumor activity of FGFR1 inhibition in SQCLC. In FGFR1 amplified/over-expressing SQCLC cell lines, FGF2-mediated stimulation of FGFR1 under serum-deprivation activated both MAPK and AKT/mTOR pathways and increased glucose uptake, glycolysis, and lactate production, through AKT/mTOR-dependent HIF-1α accumulation and up-regulation of GLUT-1 glucose transporter. These effects were hindered by PD173074 and NVP-BGJ398, selective FGFR inhibitors, as well as by dovitinib, a multi-kinase inhibitor. Glucose metabolism was hampered by the FGFR inhibitors also under hypoxic conditions, with consequent inhibition of cell proliferation and viability. In presence of serum, glucose metabolism was impaired only in cell models in which FGFR1 inhibition was associated with AKT/mTOR down-regulation. When the activation of the AKT/mTOR pathway persisted despite FGFR1 down-regulation, the efficacy of NVP-BGJ398 could be significantly improved by the combination with NVP-BEZ235 or other inhibitors of this signaling cascade, both *in vitro* and in xenotransplanted nude mice. Collectively our results indicate that inhibition of FGFR1 signaling impacts on cancer cell growth also by affecting glucose energy metabolism. In addition, this study strongly suggests that the therapeutic efficacy of FGFR1 targeting molecules in SQCLC may be implemented by combined treatments tackling on glucose metabolism.

## INTRODUCTION

The Fibroblast Growth Factor Receptor (FGFR) tyrosine kinase family contains four members (FGFR1-4), activated through 22 different FGF ligands, which regulate a variety of biological functions, including cell survival, proliferation, migration, differentiation, and angiogenesis [1]. Aberrant FGFR signaling, via amplification, point mutations or translocations, has been implicated in several human cancers [2]. Deregulated FGFR signaling can lead to cancer development and progression through multiple mechanisms that vary depending on the cellular context and the tumor type. Downstream of FGFRs, both the MAPK and PI3K/AKT pathways have been shown to mediate mitogenic signals, for example inducing Cyclin D1, as well as pro-survival signals, by upregulating the expression of anti-apoptotic proteins, such as Bcl-2, Bcl-XL and X-linked inhibitor of apoptosis (XIAP) or negatively regulating pro-apoptotic proteins such as BAD [1]. FGFR signaling can also promote cell migration and invasion. In breast cancer, FGFR1-dependent activation of STAT3 was shown to stimulate both cell proliferation and migration by increasing the synthesis of the extracellular matrix component hyaluronan [3]. In addition, FGFR-mediated induction of the epithelial-mesenchymal transition (EMT) has been associated with the acquisition of an invasive phenotype in different cancer cell models [4, 5]. A further mechanism contributing to FGFR-dependent tumorigenesis may involve the acquisition of a metabolic advantage through the induction of the Warburg effect. Indeed, FGFR1 has been shown to directly phosphorylate pyruvate kinase M2 (PKM2), a key enzyme of glycolysis, allowing its switching towards a less active dimeric form and therefore enhancing the use of glycolytic intermediates for macromolecular biosynthesis and tumor growth [6]. In addition, it has been demonstrated that FGFR1 can contribute to the Warburg effect by phosphorylating and activating the pyruvate dehydrogenase (PDH) kinase 1 (PDHK1), thus attenuating the mitochondrial function [7]. Apart from FGFR1-mediated effects on these metabolic enzymes, to our knowledge the role of FGFR1 in the modulation of glucose energy metabolism in cancer cells has not been accurately explored so far.

Squamous cell lung cancer (SQCLC) is the second most common type of lung cancer, representing approximately 30-40% of all Non Small Cell Lung Cancer (NSCLC), and is almost invariably associated with smoking; due to its mutational complexity, efficacious molecular-targeted treatments are difficult to develop and platinum-based regimens remain the standard of care 1^st^-line therapy for advanced disease. Very recently, immunotherapy has emerged as a valuable therapeutic strategy for SQCLC patients with progressive disease, with anti-PD1 (Nivolumab [8] and Pembrolizumab [9, 10]) and anti-PD-L1 (Atezolizumab [11]) agents approved in the treatment of this disease. In addition, over the last years efforts aimed at defining novel therapeutic options for the treatment of SQCLC have led to the identification of FGFR signaling as an attractive therapeutic target, being one of the most frequently altered pathways in this NSCLC subtype [12]. In particular, deregulation of FGFR signaling is associated with FGFR1 amplification through *in situ* hybridization (FISH) analysis in approximately 20% of SQCLC [13, 14], although a lower frequency (9%) emerged from more recent analyses based on next generation sequencing [15].

The present study was designed to investigate the role of FGFR1 signaling in the regulation of glucose energy metabolism in FGFR1 amplified/over-expressing SQCLC models showing different patterns of molecular alterations. We demonstrated that FGFR1 actually controls glucose uptake and utilization by activating the AKT/mTOR pathway, which in turn is responsible for the induction of HIF-1α and GLUT-1 glucose transporter expression, under both normoxic and hypoxic conditions. In addition, FGFR inhibitors - NVP-BGJ398 and PD173074, with selectivity against FGFRs, and dovitinib (TKI258), targeting also Vascular Endothelial Growth Factor Receptors (VEGFRs), Platelet Derived Growth Factor Receptors (PDGFRs), FLT3 and c-Kit [16] - were shown to exert anti-tumor activity by hampering glucose metabolism through AKT/mTOR inhibition. Moreover, our data suggest that the combination of selective FGFR inhibitors with targeted down-regulation of AKT/mTOR signaling pathway and hence glucose utilization was able to improve the therapeutic efficacy of FGFR inhibition both *in vitro* and *in vivo*.

## RESULTS

### Effects of FGFR1 inhibition on FGFR1-amplified H1703 and H520 cells in the presence of serum

**Figure 1:**
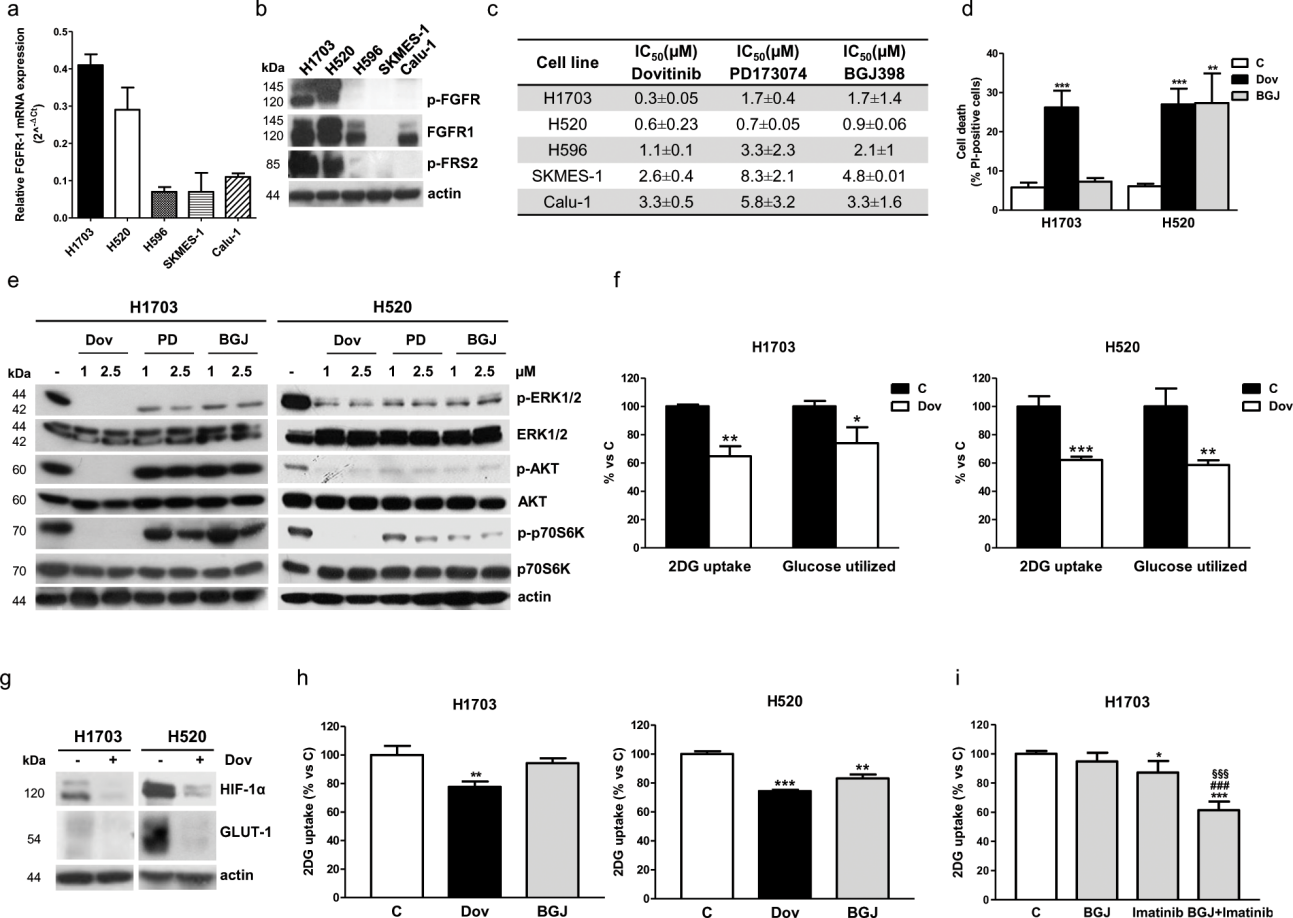
Effects of FGFR1 inhibition in FGFR1-amplified H1703 and H520 cells in normal growth conditions **(a)** SQCLC cells incubated in normal growth conditions were analyzed for FGFR1 mRNA expression by RT-PCR. Data are expressed as relative FGFR1 mRNA expression (2^-ΔCT ± SD). **(b)** Protein extracts from SQCLC cells were analyzed by Western Blotting for the expression of the indicated proteins. **(c)** Cells were treated with or without increasing concentrations of dovitinib, PD173074, or NVP-BGJ398 (0.01-10μM). After 72h cell survival/proliferation was assessed using CV assay. The IC_50_ values shown are mean ±SD of three independent experiments. **(d)** H1703 and H520 cells were treated with or without 1μM dovitinib or NVP-BGJ398. After 72h cell death was assessed on Hoechst 33342/PI stained cells. **(e)** H1703 and H520 cells were exposed to the FGFR inhibitors for 24h, and then protein extracts were analyzed by Western Blotting for the expression of the indicated proteins. **(f)** H1703 and H520 cells were incubated with 1μM dovitinib for 24h and then glucose uptake and glycolysis were measured. Data are expressed as percent versus control cells (C). **(g)** The cells were treated as in **(f)** and protein lysates were analyzed by Western Blotting for HIF-1α and GLUT-1 expression. **(h)** H1703 and H520 cells were incubated with 1μM dovitinib or NVP-BGJ398. Glucose uptake was measured after 6h. Data are expressed as percent versus control cells (C). **(i)** H1703 cells were treated with 1μM NVP-BGJ398, 1μM imatinib mesylate or a combination of both. Glucose uptake was measured after 24h. Data are expressed as percent versus control cells (C). Results in **(a, b, e**, and **g)** are representative of three independent experiments. Data in **(d, f, h**, and **i)** are mean values ±SD of three independent experiments. ^*^P<0.05, ^**^P<0.01, ^***^P<0.001 vs C; ^###^P<0.001 vs BGJ; ^§§§^P<0.001 vs imatinib.

We investigated the role of FGFR1 signaling in FGFR1-amplified H1703 and H520 SQCLC cells [17]. These cell lines expressed the highest levels of FGFR1 mRNA and protein in comparison with other SQCLC cell models without FGFR1 amplification (H596, SKMES-1, and Calu-1), and showed a significant phosphorylation of both FGFR1 and the downstream adapter protein FRS-2, indicating the presence of a functional receptor signaling (Figure 1a, 1b). H1703 cells were more sensitive to the multi-kinase inhibitor dovitinib in comparison with PD173074 or NVP-BGJ398 selective FGFR inhibitors (Figure 1c, 1d) presumably due to the amplification of the PDGFRα receptor [18], an additional target of dovitinib. These results confirmed previous data from literature [19], suggesting that the response to FGFR1 inhibition in SQCLC cells not only depends on the expression of the FGFR1 receptor, but is also affected by the contemporary activation of alternative signaling pathways that may contribute to tumor cell growth.

The divergent activity of the FGFR inhibitors on cell proliferation and death was associated with different effects on intracellular signaling pathways. Indeed, dovitinib completely inhibited both the MAPK and AKT/mTOR pathways in H1703 cells (an effect detectable also at 0.5μM, not shown) in contrast with PD173074 and NVP-BGJ398, which failed to down-regulate the AKT/mTOR pathway (Figure 1e), suggesting that in these cells the activation of this signaling cascade may be controlled not only by FGFR1 but also by PDGFRα. On the contrary, in H520 cells dovitinib and the selective FGFR inhibitors down-regulated both the MAPK and AKT/mTOR pathways (Figure 1e) and accordingly produced comparable growth inhibitory effects (Figure 1c, 1d).

We then evaluated the effects of FGFR1 inhibition on glucose metabolism and demonstrated that dovitinib treatment promoted a significant decrease of glucose uptake and glycolysis, associated with the down-regulation of hypoxia-inducible factor-1α (HIF-1α) and glucose transporter 1 (GLUT-1) expression, in both H1703 and H520 cells (Figure 1f, 1g). In contrast, NVP-BGJ398 down-regulated the glucose uptake in H520 cells, but was ineffective in H1703 cells (Figure 1h). The same result was obtained with PD173074 (not shown), suggesting that in H1703 cells serum-mediated activation of the PDGFRα pathway can mask the effects of the selective inhibition of FGFR1 signaling. Indeed, inhibition of PDGFRα by imatinib down-regulated the glucose uptake; however, only when imatinib was combined with NVP-BGJ398 a marked reduction, comparable to that induced by dovitinib treatment, was achieved, indicating that both PDGFRα and FGFR1 contribute to the modulation of glucose metabolism in H1703 cells (Figure 1i). Collectively these results also indicate that the AKT/mTOR pathway may be involved in the regulation of glucose metabolism in SQCLC cells.

### Effects of FGFR1 inhibition under FGF2 stimulation in serum-deprived conditions

To clarify the role of FGFR signaling and the effects of its inhibition in SQCLC cells, we performed experiments under serum-deprivation stimulating the cells with FGF2, physiological ligand of FGFR1, to exclude the stimuli mediated by other serum components.

**Figure 2:**
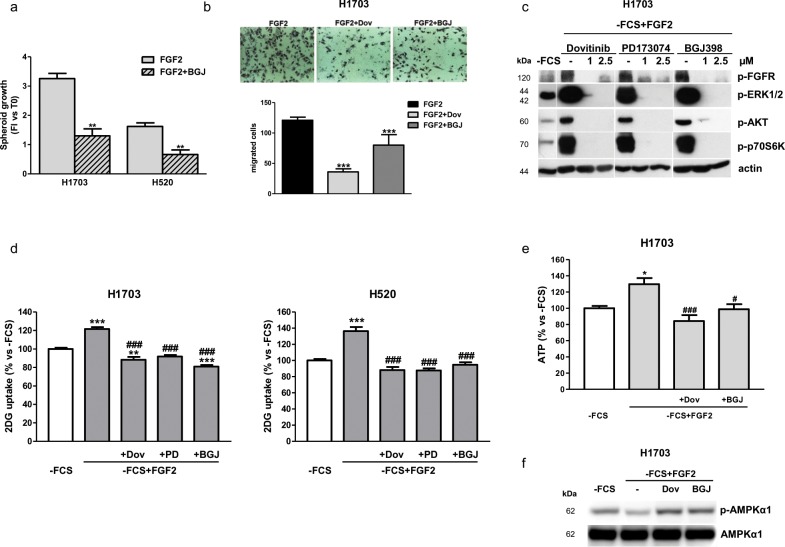
Effects of FGFR1 inhibition under FGF2 stimulation in serum-deprived H1703 and H520 cells **(a)** The growth of spheroids from H1703 and H520 cells was analyzed after 7 days of treatment with 1μM NVP-BGJ398 in the presence of FGF2. Data are expressed as Fold Increase (FI) index, calculated as the ratio between the spheroid volume after 7 days and the volume at T0. ^**^P<0.01 vs FGF2-treated cells. **(b)** Cell migration assay was performed in H1703 cells incubated in 1% FCS-containing medium in the presence or absence of 1μM dovitinib or NVP-BGJ398, with FGF2 used as chemoattractant. Representative fields of migration are shown (magnification of 100X). Data in columns are means ±SD of 10 fields counted. ^***^P<0.001 vs FGF2-treated cells. **(c)** H1703, deprived of serum (-FCS) for 24h, were pre-incubated for 1h with 1μM dovitinib, PD173074, or NVP-BGJ398 and stimulated with FGF2 for further 15min. Then protein expression was assessed by Western Blotting. **(d)** H1703 and H520 cells, incubated in -FCS for 24h, were pre-incubated for 1h with the FGFR inhibitors at 1μM and then treated with FGF2. Glucose uptake was measured after 6h. Data are expressed as percent versus -FCS control cells. **(e)** H1703, incubated in-FCS for 24h, were pre-incubated for 1h with 1μM dovitinib or NVP-BGJ398 and then treated with FGF2. After 24h ATP intracellular levels were measured by a luminescence assay. Data are expressed as percent versus -FCS control cells. **(f)** H1703 cells were treated as in **(e)**. After 24h cell protein lysates were analyzed by Western Blotting for p-AMPKα1 and total AMPKα1 expression. Results in **(b, c**, and **f)** are representative of at least two independent experiments. Data in **(a, d**, and **e)** are mean values ±SD of three independent experiments. ^*^P<0.05, ^**^P<0.01, ^***^P<0.001 vs -FCS; ^#^P<0.05, ^###^P<0.001 vs -FCS+FGF2.

In this condition, NVP-BJG398 treatment was capable of inhibiting FGF2-mediated cell proliferation and growth in H1703 cells as well as in H520 cells, in both two dimensional (2D, not shown) and three dimensional (3D) systems (Figure 2a). In addition, FGF2 treatment promoted cell migration in H1703 cells, and both dovitinib and NVP-BJG398 were effective in inhibiting this process (Figure 2b). As shown in Figure 2c, FGF2 stimulation of H1703 cells greatly induced both MAPK and AKT/mTOR signaling which were down-regulated by serum-deprivation; treatment with dovitinib, PD173074, and NVP-BGJ398 inhibited both signaling pathways, as indicated by the almost complete dephosphorylation of their main components. Interestingly, FGF2-mediated activation of FGFR1 significantly induced the glucose uptake, which was prevented not only by dovitinib but also by the selective inhibitors in H1703 and H520 cells (Figure 2d). In addition, FGFR1 inhibition prevented the ATP production associated with FGF2 stimulation (Figure 2e). Reduced ATP levels resulted in the phosphorylation/activation of 5′ AMP-activated protein kinase (AMPK), a master energy sensor activated by increased intracellular AMP:ADP ratio (Figure 2f).

### Effects of modulation of FGFR1 expression in FGFR1-amplified H520 cells and in FGFR1 low-expressing SKMES-1 cells

**Figure 3:**
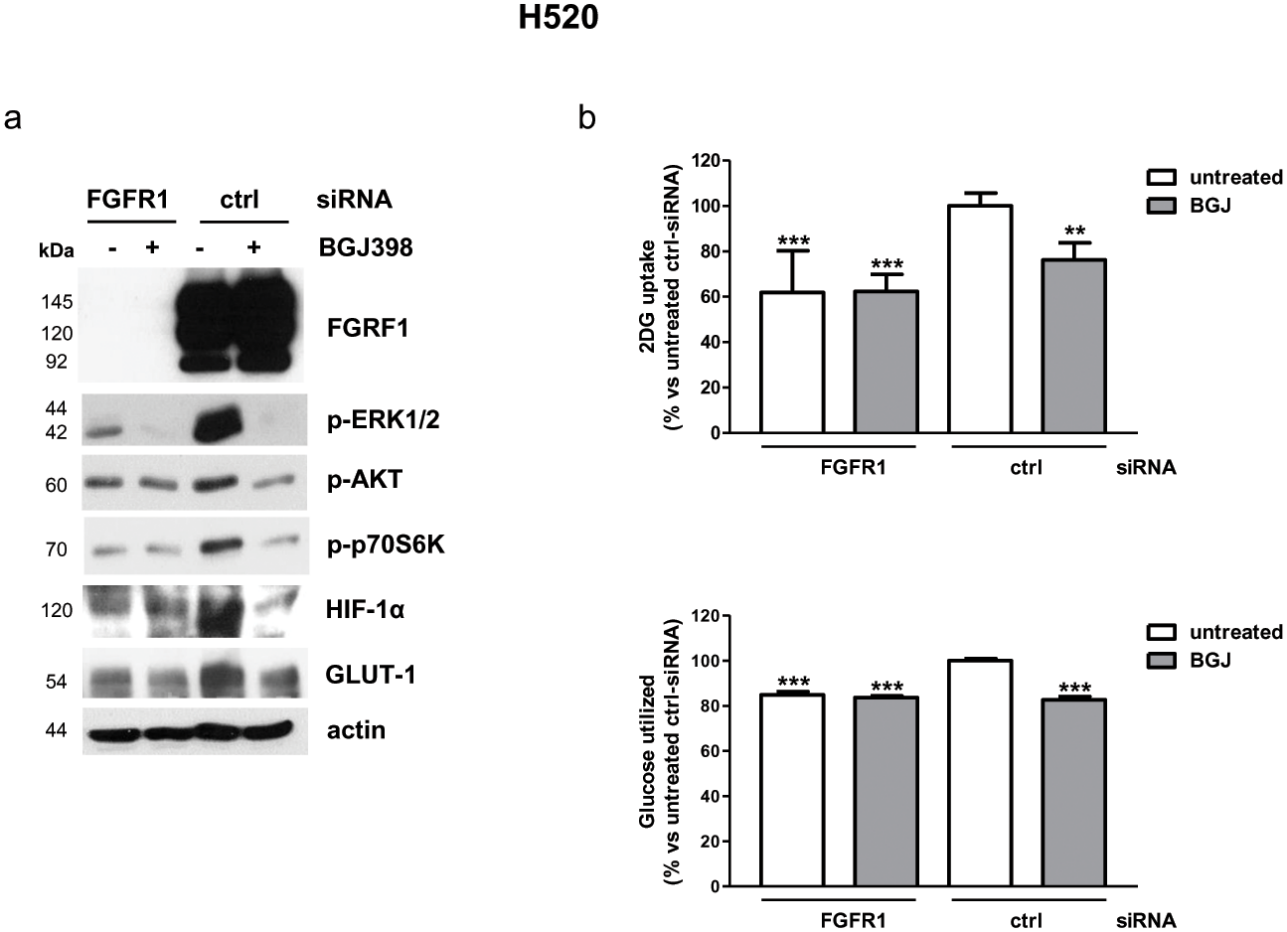
Effects of FGFR1 silencing on intracellular pathways and glucose utilization in H520 cells H520 cells were treated with a pool of FGFR1 siRNA or control siRNA for 48h and then treated with 1μM NVP-BGJ398 for 16h. **(a)** Cell protein extracts were analyzed by Western Blotting for the indicated proteins. Results are representative of three independent experiments. **(b)** Glucose uptake and glycolysis were measured. Data are mean values ±SD of three independent experiments and are expressed as percent versus untreated control siRNA. ^**^P<0.01, ^***^P<0.001 vs untreated control siRNA.

To mimic the effects of receptor selective inhibition, H520 cells were treated with a pool of specific FGFR1-targeting siRNAs, that completely abolished the receptor expression (Figure 3a). FGFR1 silencing significantly reduced cell proliferation, increasing the percentage of cells in the G_0/1_ phase of the cell cycle (Supplementary Figure 1a, 1b), a result in accordance with previous studies showing that transduction of H520 cells with lentivirus encoding FGFR1-targeting shRNAs suppressed colony outgrowth [17]. In this condition, both MAPK and AKT/mTOR pathways, together with HIF-1α and GLUT-1 protein expression, were down-regulated (Figure 3a), and a significant reduction of glucose uptake was observed, which was associated with a decrease of glucose utilization through glycolysis (Figure 3b). NVP-BGJ398 treatment of FGFR1-silenced cells yielded results similar to NVP-BGJ398 or siRNA-FGFR1 treatments alone, indicating that the drug inhibitory effects actually relied on FGFR1 inhibition.

**Figure 4:**
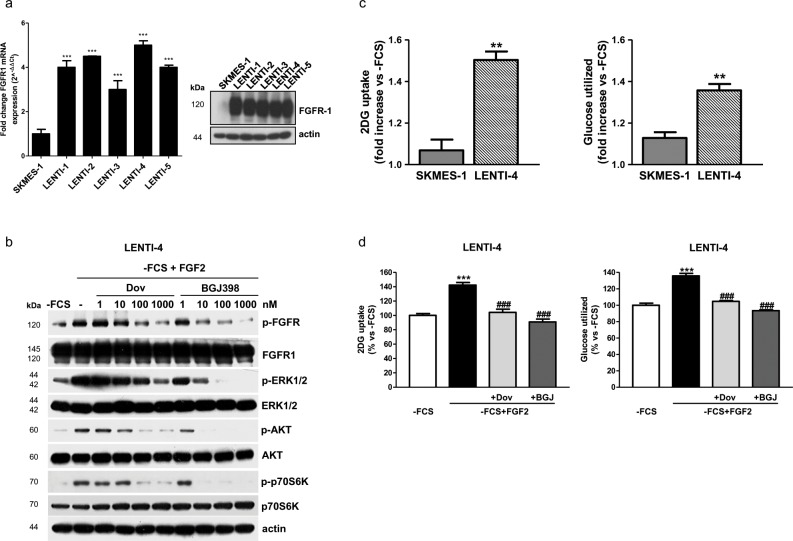
Effects of FGFR1 inhibition in FGFR1 over-expressing SKMES-1-derived cells **(a)** SKMES-1 parental cells and FGFR1-overexpressing cells were analyzed for FGFR1 mRNA expression by RT-PCR. Data are expressed as mRNA quantity normalized to SKMES-1 cell line (=1). ^***^P<0.001 vs SKMES-1 cells. Cell protein extracts were analyzed by Western Blotting for the expression of FGFR1 protein. **(b)** LENTI-4 cells were incubated in -FCS for 24h, then pre-incubated for 1h with increasing concentrations of dovitinib or NVP-BGJ398, and stimulated with FGF2 for further 15min. Cell protein extracts were then analyzed by Western Blotting for the indicated proteins. **(c)** SKMES-1 and LENTI-4 cells were incubated in -FCS for 24h, and then treated with FGF2. Glucose uptake and glycolysis were measured after 16h. Data are expressed as fold increase versus corresponding -FCS control cells. ^**^P<0.01 vs SKMES-1 cells. **(d)** LENTI-4 cells were incubated in -FCS for 24h, pre-incubated for 1h with 1μM dovitinib or NVP-BGJ398 and then stimulated with FGF2. Glucose uptake and glycolysis were assessed after 16h. Data are expressed as percent versus -FCS control cells. ^***^P<0.001 vs -FCS; ^###^P<0.001 vs -FCS+FGF2. Results of Western Blotting in **(a** and **b)** are representative of three independent experiments. Data in **(a, c**, and **d)** are mean values ±SD of three independent experiments.

To give strength to these results, we generated new FGFR1 over-expressing cells from SQCLC FGFR1 low-expressing SKMES-1 cells, by using a lentiviral expression vector system (Figure 4a), and LENTI-4 cells were chosen for the subsequent experiments. As shown for H1703 cells, FGF2 stimulation in LENTI-4 cells induced the phosphorylation of FGFR1 and activated both MAPK and AKT/mTOR signaling; dovitinib and NVP-BGJ398, by inhibiting FGFR1 activation, down-regulated these downstream pathways in a dose-dependent manner, with NVP-BGJ398 showing a greater inhibitory efficacy (Figure 4b). FGF2 strongly stimulated both the glucose uptake and the glycolytic flux in LENTI-4 in comparison with SKMES-1 parental cells, and again both dovitinib and NVP-BGJ398 impaired these processes (Figure 4c, 4d).

Collectively, these data point to a role of FGFR1 signaling in the modulation of glucose energy metabolism in SQCLC cells.

### Mechanisms of modulation of FGF2-mediated glucose metabolism: role of AKT/mTOR signaling and PKM2

**Figure 5:**
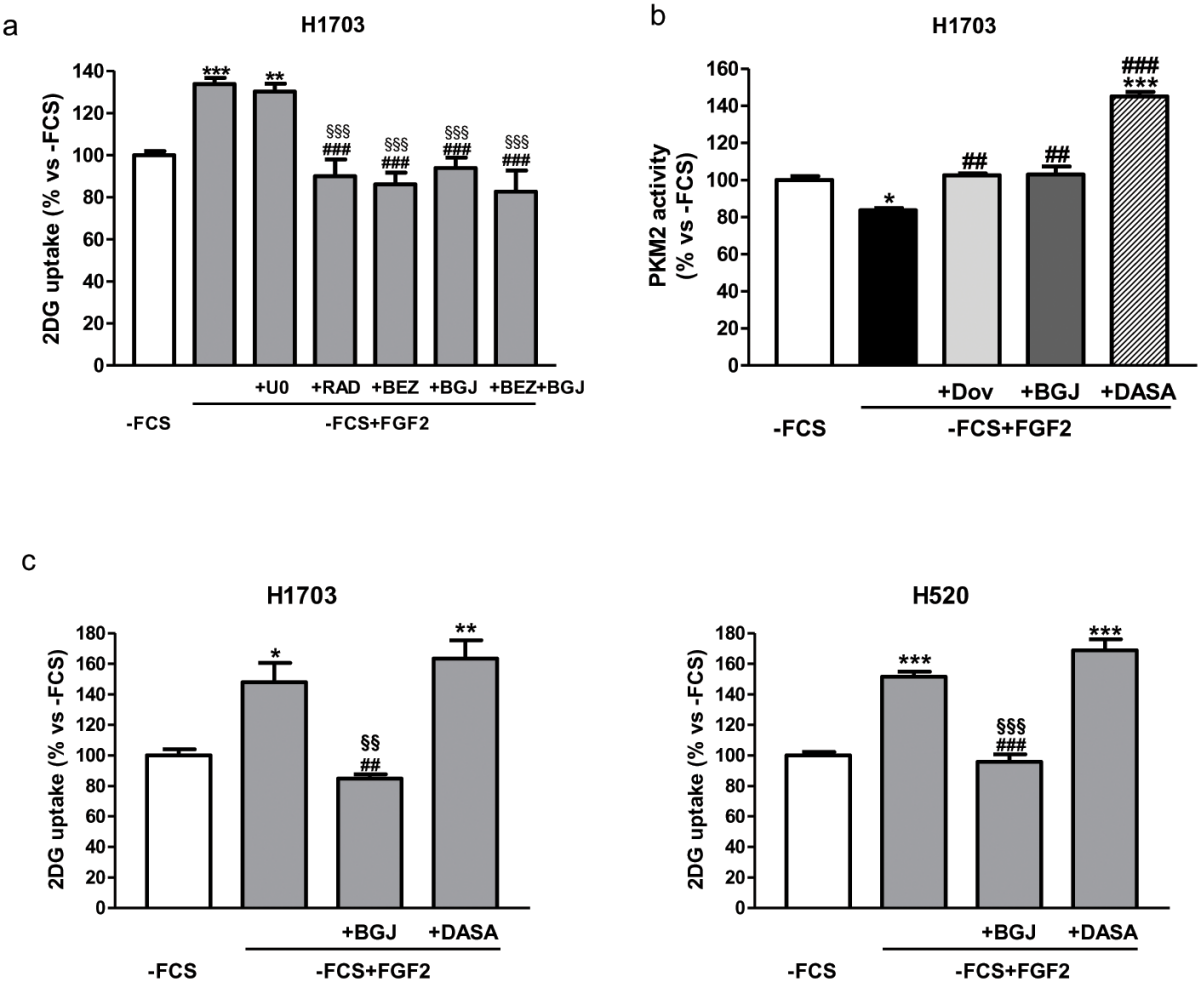
Role of AKT/mTOR and PKM2 in the regulation of FGF2-mediated glucose metabolism **(a)** H1703 cells, cultured in -FCS for 24h, were pre-incubated for 1h with 2μM U0126, 0.1μM NVP-BEZ235, 0.1μM RAD001, 1μM NVP-BGJ398 or a combination of NVP-BGJ398 with NVP-BEZ235. The cells were then stimulated with FGF2 and glucose uptake was assessed after 6h. **(b)** H1703 cells, cultured in -FCS for 24h, were pre-incubated for 1h with 1μM dovitinib, 1μM NVP-BGJ398 or 10μM DASA, and then treated with FGF2. PKM2 activity was measured after 4h. **(c)** H1703 and H520 cells, cultured in -FCS for 24h, were pre-incubated for 1h with 1μM NVP-BGJ398 or 10μM DASA, and then treated with FGF2. Glucose uptake was measured after 16h. Data are expressed as percent versus -FCS control cells and are mean values ±SD of three independent experiments. ^*^P<0.05, ^**^P<0.01, ^***^P<0.001 vs -FCS; ^##^P<0.01, ^###^P<0.001 vs -FCS +FGF2; ^§§^P<0.01, ^§§§^P<0.001 vs U0126 **(a)** or DASA **(c)**.

Since FGF2 treatment promoted a significant activation of both MAPK and AKT/mTOR signaling, efficiently prevented by the FGFR inhibitors, we sought to investigate which of the two pathways was actually involved in FGF2-mediated stimulation of glucose uptake. Serum-deprived H1703 cells were pre-treated with a highly selective MEK1-2 inhibitor (U0126), a dual PI3K/mTORC1-2 inhibitor (NVP-BEZ235), and NVP-BGJ398, and then stimulated with FGF2. As shown in Figure 5a, U0126 had no effect on glucose uptake, suggesting that the MAPK pathway did not play a role in the regulation of this process. In contrast, NVP-BEZ235, as well as NVP-BJG398, significantly hampered the glucose uptake; when NVP-BEZ235 and NVP-BJG398 were used in combination, the glucose uptake decreased to levels comparable to those observed with each agent alone, suggesting that the AKT/mTOR pathway downstream of FGFR1 is involved in the FGF2-mediated induction of glucose uptake in H1703 cells. Interestingly, selective mTOR inhibition by RAD001 was sufficient to hinder the glucose transport, pointing to a main role of mTOR in the modulation of glucose metabolism along the FGFR1 axis.

To further get insight into the mechanisms of FGFR1-mediated modulation of glucose metabolism in SQCLC cells, we evaluated the effects of FGF2 stimulation on the activity of PKM2, a key enzyme of glycolysis, that has been shown to be down-regulated by FGFR1 through direct phosphorylation [6]. As expected, FGF2-mediated activation of FGFR1 signaling in H1703 down-regulated PKM2 activity, which was, conversely, increased by treatment with dovitinib or NVP-BGJ398 (Figure 5b). To evaluate whether FGF2-dependent modulation of PKM2 activity could affect the glucose uptake, we used the PKM2 activator DASA under FGF2 stimulation. As shown in Figure 5b, DASA strongly induced PKM2 activity, overcoming the inhibitory action mediated by FGFR1 upon stimulation with FGF2; nevertheless, the uptake of glucose induced by FGF2 treatment remained unaffected in H1703 as well as in H520 cells (Figure 5c), implying that FGFR1 signaling promotes the glucose uptake independently of its effects on PKM2 activity in SQCLC cells.

### Effects of FGF2 stimulation and FGFR1 inhibition under hypoxic conditions

**Figure 6:**
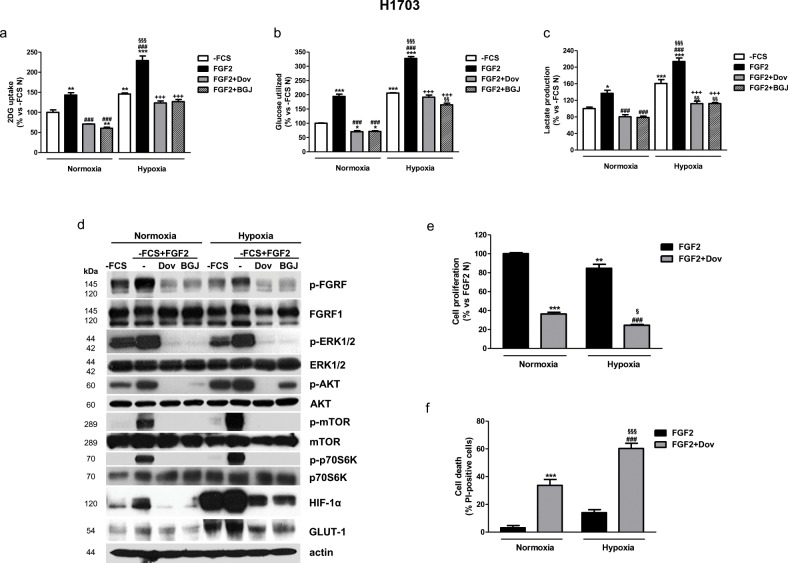
Effects of FGFR1 inhibition under FGF2 stimulation in serum-deprived H1703 cells in normoxic and hypoxic conditions H1703 cells, cultured in -FCS for 24h, were pre-incubated for 1h with 1μM dovitinib or NVP-BGJ398, stimulated with FGF2, and then incubated in normoxic and hypoxic (0.5% O_2_) conditions. After 16h glucose uptake **(a)**, glycolysis **(b)**, and lactate production **(c)** were measured and protein expression from cell lysates was assessed by Western Blot analysis **(d)**. After 48h cell proliferation **(e)** and cell death **(f)** were evaluated by cell counting with trypan blue dye exclusion method and by fluorescence microscopy on Hoechst 33342/PI stained cells, respectively. Results are mean values ±SD of three independent determinations. Data in **(a, b**, and **c)** are expressed as percent versus -FCS control cells in normoxia (-FCS N). ^*^P<0.05, ^**^P<0.01, ^***^P<0.001 vs -FCS N; ^###^P<0.001 vs FGF2 N, ^§§^P<0.01, ^§§§^P<0.001 vs -FCS Hypoxia (-FCS H), ^+++^P<0.001 vs FGF2 H. Data in **(e)** are expressed as percent versus FGF2 N. ^**^P<0.01, ^***^P<0.001 vs FGF2 N; ^###^P<0.001 vs FGF2 H; ^§^P<0.05, ^§§§^P<0.001 vs FGF2+Dovitinib N.

Since FGFR inhibitors may initiate hypoxic conditions due to their anti-angiogenic effects, we investigated the role of FGF2/FGFR1 signaling on glucose metabolism and the effects of its inhibition also under hypoxia. As shown in Figure 6a-6c, compared to the normoxic condition, hypoxia itself promoted a shift to glycolysis in H1703 cells, resulting in a significant increase of glucose uptake and lactate production. This effect was strongly enhanced by FGF2 stimulation, and prevented by both dovitinib and NVP-BGJ398, as also shown in normoxic conditions.

Treatment of H1703 cells with FGF2 led to the activation of both MAPK and AKT/mTOR pathways also under hypoxic conditions and enhanced the hypoxia-mediated induction of HIF-1α and GLUT-1 expression (Figure 6d). Interestingly, HIF-1α and GLUT-1 protein expression was induced by FGF2 also in normoxic conditions. The FGFR inhibitors down-regulated the expression of these proteins, confirming their involvement in the modulation of FGF2-dependent glucose utilization. Together these results suggest that the FGFR inhibitors, by hampering glucose metabolism, may render H1703 cells unable to adapt to hypoxia, as indicated by the observation that also this condition induced a block of cell proliferation associated with a significant increase of cell death (Figure 6e, 6f). Comparable results, confirming the role of FGFR1 signaling in the regulation of glucose metabolism under normoxia and hypoxia, were obtained in the NSCLC large cell carcinoma H1581 cell line, a cell model that harbors focal amplification of FGFR1 and is highly sensitive to FGFR1 inhibition (not shown). By contrast, in SKMES-1 cells neither FGF2 stimulation nor treatment with FGFR1 inhibitors produced relevant effects on glucose uptake under normoxic and hypoxic conditions (Supplementary Figure 2).

### Effects of NVP-BGJ398 in combination with inhibitors of PI3K/AKT/mTOR signaling *in vitro*

**Figure 7:**
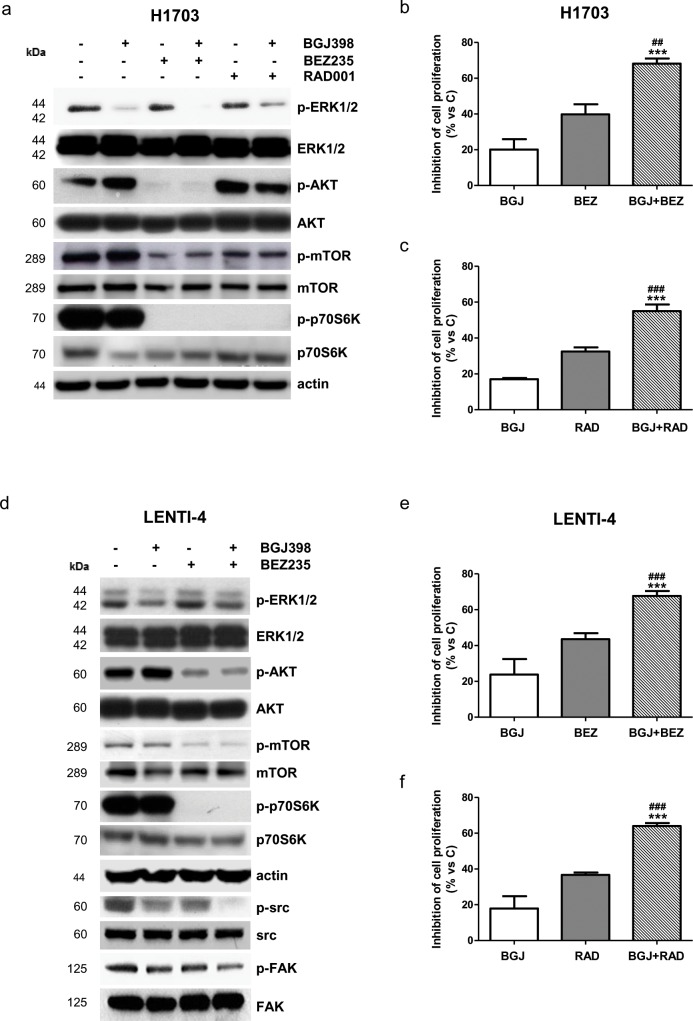
Effects of the combination of NVP-BGJ398 with AKT/mTOR inhibitors on H1703 and LENTI-4 cells **(a)** H1703 cells were treated with 1μM NVP-BGJ398, 0.1μM NVP-BEZ235, and 0.1μM RAD001 alone or with NVP-BGJ398 in combination with NVP-BEZ235 or RAD001. After 24h protein expression was assessed by Western Blot analysis. H1703 cells were treated with 1μM NVP-BGJ398 or 0.01μM NVP-BEZ235 alone or in combination **(b)** and with 1μM NVP-BGJ398 or 0.01μM RAD001 alone or in combination **(c)**. After 72h cell survival/proliferation was evaluated by CV assay. **(d)** LENTI-4 cells were treated with 1μM NVP-BGJ398 or 0.1μM NVP-BEZ235 alone or in combination. After 24h protein expression was assessed by Western Blot analysis. LENTI-4 cells were treated with 1μM NVP-BGJ398, 0.01μM NVP-BEZ235 alone or in combination **(e)** and with 1μM NVP-BGJ398 or 0.01μM RAD001 alone or in combination **(f)**. After 72h cell survival/proliferation was evaluated by CV assay. Results in **(a** and **d)** are representative of three independent experiments. Data in **(b, c, e**, and **f)** are expressed as percent inhibition of cell proliferation vs control cells (C) and are mean values ±SD of three independent experiments. ^***^P<0.001 vs NVP-BGJ398; ^##^P<0.01, ^###^P<0.001 vs NVP-BEZ235 or RAD001.

Having demonstrated the requirement for the AKT/mTOR signaling inhibition to achieve a down-regulation of glucose utilization and hence a better anti-proliferative response in NSCLC cells (see effects of dovitinib versus selective FGFR inhibitors in H1703 cells), we hypothesized that NVP-BGJ398 treatment might be conveniently associated with inhibitors of the PI3K/AKT/mTOR pathway in those cell models in which FGFR1 inhibition is not sufficient to inhibit this signaling cascade in the presence of serum. Actually, in H1703 cells the combination of NVP-BGJ398 with the dual PI3K/mTORC1-2 inhibitor NVP-BEZ235 (Figure 7a) or the PI3K inhibitor NVP-BKM120 (not shown), down-regulating both the MAPK and the AKT/mTOR pathways, induced a stronger inhibition of cell proliferation in comparison with single agents alone (Figure 7b). Remarkably, comparable effects were produced combining NVP-BGJ398 with the selective mTORC1 inhibitor RAD001, despite AKT phosphorylation was increased due to the release of mTOR-dependent feedback inhibition of IRS-1/PI3K/AKT signaling (Figure 7a, 7c). Combinations of NVP-BGJ398 with PI3K/mTOR inhibitors inhibited cell proliferation more efficaciously than single agents also in FGFR1 over-expressing LENTI-4 cells. Interestingly, NVP-BGJ398 reduced the phosphorylation/activation of src and its target focal adhesion kinase (FAK), and this inhibitory effect was further potentiated by the combination with NVP-BEZ235 (Figure 7d-7f). Down-regulation of this signaling cascade, together with NVP-BEZ235-mediated inhibition of the AKT/mTOR pathway, presumably contributed to the enhanced anti-proliferative activity of the drug combination in LENTI-4 cells.

### Effects of NVP-BGJ398 combined with NVP-BEZ235 in LENTI-4 tumor xenografts

**Figure 8:**
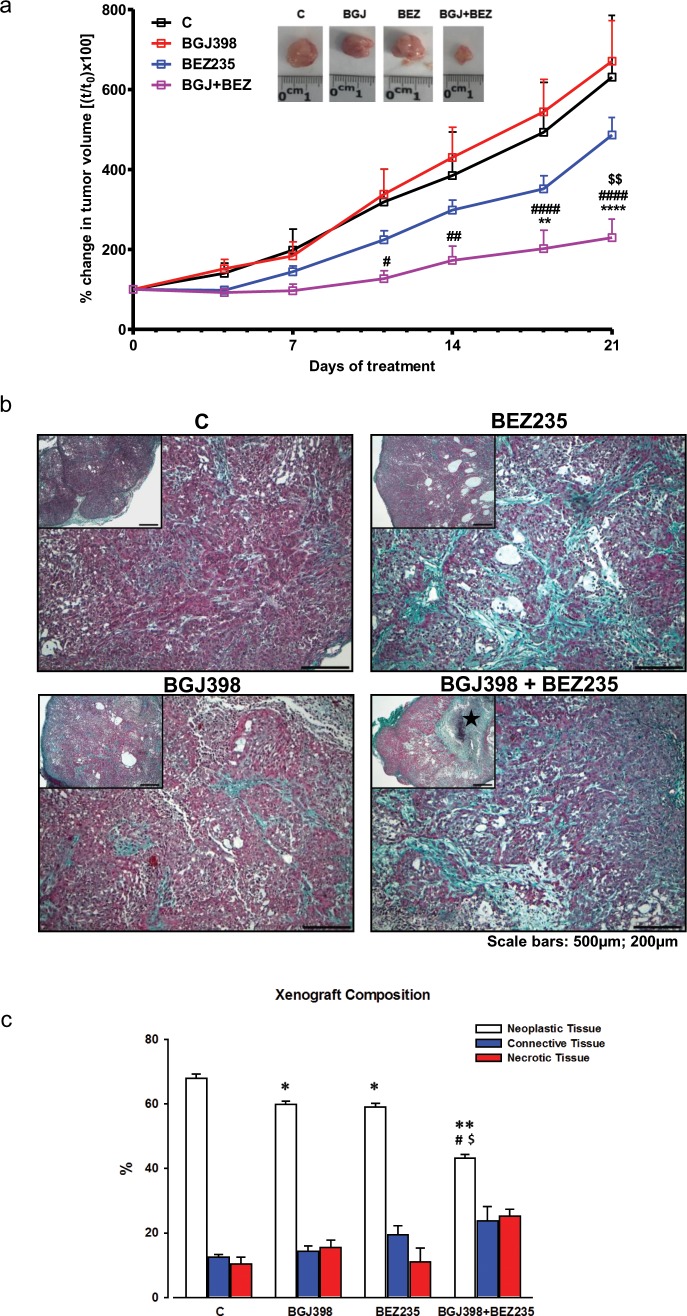
*In vivo* effects of NVP-BGJ398 and NVP-BEZ235 on LENTI-4 tumor xenografts LENTI-4 cells were implanted s.c. in BALB/c-Nude mice. Vehicle, NVP-BGJ398 (30 mg/Kg) and NVP-BEZ235 (15 mg/kg) were administered five times per week by orogastric gavage. **(a)** Tumor sizes were measured two times per week and data are expressed as percentage of change in tumor volume ± SEM of 8 tumors per group. ^**^p<0.01, ^****^p<0.0001 vs C; ^#^p<0.05, ^##^p <0.01, ^####^p<0.0001 vs NVP-BGJ398; ^$$^p<0.01 vs NVP-BEZ235. Inset: representative images of dissected xenograft tumors. **(b)** Panel Insets: low magnifications of selected examples of Masson's Trichrome stained sections of subcutaneous LENTI-4 induced tumor xenograft from untreated (C) and drug treated mice. ★ in NVP-BGJ398+NVP-BEZ235 indicates a large necrotic area (scale bars: 500μm). Representative microscopic images of the same samples are shown at higher magnification on corresponding panels. Intense collagen deposition (greenish) between neoplastic cells (purple) is apparent in NVP-BEZ235 and NVP-BGJ398+NVP-BEZ235 treated xenografts (scale bars: 200μm). **(c)** Bar graph illustrating the quantitative measurements of neoplastic, connective and necrotic tissue compartments composing LENTI-4 induced tumor xenografts from untreated (C) and drug treated mice. ^*^p<0.05, ^**^p<0.01 vs C; ^#^p<0.05 vs NVP-BGJ398; ^$^p < 0.05 vs NVP-BEZ235.

To confirm the efficacy of combining NVP-BGJ398 with NVP-BEZ235 *in vivo*, we generated SQCLC xenografts in athymic mice by s.c. injection of LENTI-4 cells. After tumors had reached an average volume of about 200mm^3^, the animals were randomized into four different groups: control (C), NVP-BGJ398 (30 mg/Kg), NVP-BEZ235 (15 mg/kg) and NVP-BGJ398 plus NVP-BEZ235. Tumor growth was monitored for three weeks and during this period mice showed no signs of toxicity and regularly gained body weight (not shown). As shown in Figure 8a, the combination of NVP-BGJ398 with the dual AKT/mTOR inhibitor significantly inhibited tumor growth in comparison with single drug treatments.

**Figure 9:**
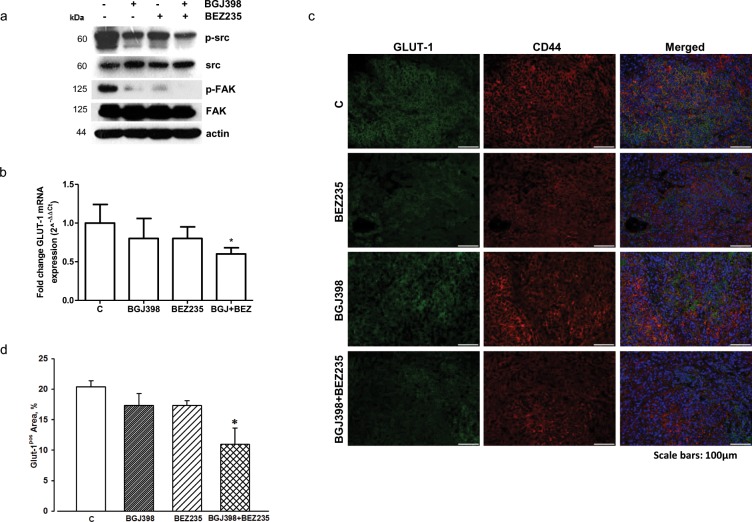
Effects of NVP-BGJ398 and NVP-BEZ235 on src/FAK signaling and GLUT-1 expression in LENTI-4 tumor xenografts **(a)** Total proteins were extracted from tissue samples obtained from LENTI-4 induced tumor xenografts from control and NVP-BEZ235, NVP-BGJ398 or NVP-BGJ398+NVP-BEZ235 treated BALB/c-Nude mice. Western Blotting was performed to evaluate src and FAK activation/expression. Results are representative of two independent experiments. **(b)** Total RNA was isolated from tissue samples obtained as in **(a)**. Human GLUT-1 mRNA levels were analyzed by RT-PCR. Amplifications were normalized to HPRT1 and PGK1. Fold changes were calculated by the ΔΔCT method and results were plotted as 2^-ΔΔCT ± SD. ^*^p<0.05 vs C. **(c)** Immunofluorescence analysis of GLUT-1 protein expression in sections of tumors from controls (C) and NVP-BEZ235, NVP-BGJ398 or NVP-BGJ398+NVP-BEZ235 treated mice. Neoplastic cells are labelled by CD44 (red) and green fluorescence documents the expression of GLUT-1. In merged images, yellowish fluorescence corresponds to CD44pos tumor cells expressing GLUT-1 while bright red fluorescence indicates GLUT-1 negative cells that appear abundant in NVP-BGJ398+NVP-BEZ235 xenograft. Scattered clusters of GLUT-1pos/CD44neg cells are also present. Nuclei are stained by the blue fluorescence of DAPI. Scale bars: 100μm. **(d)** Bar graph of the quantitative analysis of the fractional area occupied by GLUT-1 positive cells; ^*^ p<0.05 vs C.

We assessed the real impact of the different pharmacologic treatments on tumor mass by accurate morphometric analysis of tissue composition within the nodule. By this approach, a significant reduction in the fractional volume occupied by neoplastic cells was documented in xenografts after the administration of NVP-BGJ398 (-12.10%) or NVP-BEZ235 (-13.23%) when compared to control group. The simultaneous inhibition of FGFR1 by NVP-BGJ398 and PI3K/mTORC1-C2 by NVP-BEZ235 resulted in a nearly 40% decrease in neoplastic tissue when compared to control group and by 27.7% and 26.8% when compared to individual NVP-BGJ398 or NVP-BEZ235 treatments, respectively (Figure 8b, 8c). Interestingly, as shown by Western Blot analysis performed on tissue tumor extracts, the combination of NVP-BGJ398 and NVP-BEZ235 inhibited the src/FAK signaling pathway, confirming the result obtained *in vitro* (Figure 9a). In addition, RT-PCR analysis demonstrated that also GLUT-1 mRNA expression was significantly down-regulated by the combined treatment (Figure 9b). The expression of GLUT-1 was also assessed by immunohistochemistry on treated and untreated tumor xenografts. Compared to controls, a significant 46.29% reduction in GLUT-1 positive cells was documented in tumors treated with the combination of NVP-BGJ398 and NVP-BEZ235 while the inhibitory effect of individual drugs did not reach statistical significance (Figure 9c, 9d).

## DISCUSSION

Altered glucose metabolism is considered as an important hallmark of cancer. The switch towards an energy metabolism largely based on glycolysis, even in the presence of oxygen, leads to a metabolic state termed “aerobic glycolysis”, also known as the Warburg effect. Currently, the data available on the involvement of FGFR1 signaling in cancer glucose metabolism concern the enzyme PKM2, which catalyzes the final step of the glycolytic pathway, the conversion of phosphoenolpyruvate and ADP to pyruvate and ATP. FGFR1 can directly phosphorylate PKM2 at tyrosine residue 105 promoting the shift from an active tetrameric form to a less active dimeric form. This has been shown to increase the availability of glycolytic metabolites for other biosynthetic processes, thereby supporting the rapid growth of cancer cells [6]. In addition, FGFR1 can phosphorylate and activate the mitochondrial PDHK1, which in turn inactivates the pyruvate dehydrogenase (PDH) and hence the pyruvate dehydrogenase complex (PDC), reducing the entry of pyruvate into the TCA cycle and attenuating the mitochondrial function [7].

In the present study we demonstrate that an additional mechanism contributing to FGFR1-dependent modulation of glucose metabolism in SQCLC cells involves the activation of the AKT/mTOR pathway, which in turn is responsible for the induction of HIF-1α and GLUT-1 expression, under both normoxic and hypoxic conditions.

To investigate the role of FGFR1 in the modulation of glucose metabolism in SQCLC, firstly we used two FGFR1-amplified cell models, H1703 and H520 cells, showing a different dependence on FGFR1 signaling for their growth and a different sensitivity to FGFR inhibitors. In particular, the multi-kinase inhibitor dovitinib inhibited cell growth and induced cell death in both cell lines, through down-regulation of MAPK and AKT/mTOR pathways. In contrast, the selective FGFR inhibitors PD173074 and NVP-BGJ398 were less effective in H1703 cells, due to the amplification of PDGFRα receptor, which in normal culture conditions maintained the AKT/mTOR pathway activated despite FGFR1 inhibition. These observations confirm previous studies showing that co-activation of tyrosine kinase receptors may interfere with the efficacy of FGFR inhibitors [19]. Data from literature suggest that MAPK pathway is the major intracellular signaling pathway activated in FGFR1-amplified cells [13, 19]. On the other hand, it has been reported that inhibition of FGFR signaling by NVP-BGJ398 results in a transient dephosphorylation of ERK1/2 and is associated with a persistent down-regulation of AKT phosphorylation, pointing to AKT and not ERK as a valuable pharmacodynamic biomarker for NVP-BGJ398 [20]. Therefore, the effects of FGFR inhibitors on intracellular signaling pathways may vary on the different experimental growth conditions, as the incubation time and drug concentrations. Here we show that when H1703 cells were exposed to FGF2 stimulation under serum-deprivation, to exclude potential confounding factors, not only MAPK but also AKT/mTOR were activated downstream of FGFR1, and this phenomenon was efficaciously prevented by both dovitinib and selective FGFR inhibitors. Under this condition, we investigated the role of FGFR1 signaling on glucose energy metabolism and demonstrated that FGF2 induced the expression of HIF-1α and GLUT-1 proteins and significantly enhanced glucose uptake, glycolysis, and lactate production; treatment with dovitinib and NVP-BJG398 hindered all these processes. Interestingly, both FGFR inhibitors prevented the FGF2-mediated increase of intracellular ATP, causing the activation of the energy sensor AMPK. Since persistent activation of AMPK is known to down-regulate mTOR signaling under conditions of energetic stress [21, 22], we cannot rule out its contribution to maintain mTOR suppressed in the presence of FGFR inhibitors. In this context it is worth noting that AMPK has been shown to negatively regulate aerobic glycolysis in cancer cells by inhibiting HIF-1α stabilization, pointing to a role for this kinase as a tumor suppressor [21]. The stimulatory action of FGF2 and the negative effects of FGFR inhibitors on glucose metabolism were also observed in H520 cells. In addition, FGFR1 silencing in these cells down-regulated both AKT/mTOR and MAPK pathways and reduced HIF-1α and GLUT-1 protein expression, leading to a significant decrease of glucose uptake and glycolysis. The involvement of FGF2/FGFR1 signaling in the modulation of glucose metabolism was further confirmed in LENTI-4 cells, a FGFR1 over-expressing cell model generated in our lab from SQCLC SKMES-1 cells using a lentiviral expression vector.

A master regulator of glucose metabolism in cancer cells is the PI3K/AKT/mTOR pathway [23, 24]. AKT has been shown to promote the metabolic shift towards aerobic glycolysis through a variety of mechanisms, including the phosphorylation/activation of key glycolytic enzymes, such as hexokinase II and phosphofructokinase 2, and the induction of glucose transporters expression and their localization to cell membrane [25]. AKT may also indirectly affect glucose metabolism, through its downstream target mTOR, that possesses a well-recognized role as a crucial sensor of metabolic and environmental clues, integrating mitogenic signals with nutrient availability. Among its multiple functions, mTOR may favor cancer metabolic reprogramming by activating HIF-1α even under normoxic conditions. In turn, HIF-1α enhances glycolysis, increasing the transcription of genes encoding glucose transporters and glycolytic enzymes [26]. Also ERK1/2, along the MAPK pathway, has been demonstrated to enhance the transcriptional activity of HIF-1α through direct phosphorylation [27, 28]. In our experimental system, the FGFR1 downstream AKT/mTOR pathway and not the MAPK pathway seems to play a major role in the modulation of glucose metabolism. This contention is supported by the observation that the PI3K/mTORC1-2 inhibitor NVP-BEZ235, and not the MAPK inhibitor U0126, was capable of inhibiting the FGF2-stimulated glucose uptake. Most notably, also the selective inhibition of mTOR by RAD001 resulted in the down-regulation of this process, suggesting that mTOR may be sufficient to control FGFR1-mediated glucose uptake and utilization, presumably through a mechanism involving HIF-1α-dependent induction of GLUT-1 transporter. All these conclusions were further reinforced by the observation that in the presence of serum, and hence of stimuli other than FGF2, NVP-BGJ398 treatment impaired glucose metabolism only in cell lines in which FGFR1 selective inhibition resulted also in the down-regulation of mTOR signaling.

FGF2-mediated increase of glucose metabolism in H1703 cells was associated with a down-regulation of PKM2 activity. Conversely, treatment with dovitinib and NVP-BJG398 hindered the glucose utilization and increased PKM2 activity, by relieving FGFR1-mediated PKM2 phosphorylation and shift towards the dimeric form. Treatment with the PKM2 activator DASA did not affect the increased glucose uptake promoted by FGF2 either in H1703 or in H520 cells, suggesting that FGF2/FGFR1 signaling modulates this process independently of the effects on PKM2 activity. However, we cannot rule out a contribution of PKM2 to FGF2-stimulated glucose utilization independently of its catalytic activity as a glycolytic enzyme. Indeed, PKM2 has been shown to act also as a transcriptional activator of HIF-1α, which in turn mediates the transcription of several genes involved in glucose metabolism [29].

Apart from oncogene-mediated regulation of HIF-1α activity, this transcriptional factor is known for its key role in the response to low oxygen concentrations. Under hypoxia, hydroxylation and subsequent polyubiquitination of HIF-1α protein are restricted, promoting its accumulation and increased transcriptional activity. Conversely, mTOR function is negatively regulated as a strategy to conserve energy [30]. However, in H1703 cells exposed to hypoxia under serum-deprivation, FGF2 stimulation strongly induced mTOR phosphorylation/activation, and the expression of HIF-1α protein, induced by hypoxia, was further increased, enhancing glucose utilization through glycolysis. In this context, it is worth noting that a positive feedback loop in which HIF-1α and FGF2 mutually amplify their expression has been described as a mechanism contributing to the angiogenic response under hypoxia in different experimental systems [31, 32]. In our study, treatment with FGFR inhibitors in FGF2-stimulated H1703 cells down-regulated the signal through the FGFR1/AKT/mTOR/HIF-1α axis, and impeded the metabolic adaptation to hypoxia, resulting in a significant decrease of cell proliferation and viability. Similar results were obtained in H1581 cells. These data may have clinical relevance, considering that hypoxia is a common microenvironmental condition of solid tumors, including NSCLC, and that the angiogenic action of FGFR inhibitors may further increase the cell dependence on hypoxia adaptations. Accordingly, approaches targeting HIF-mediated metabolic adaptations in combination with anti-angiogenic therapies have proved superior efficacy in pre-clinical models and have been proposed as valuable clinical strategies to improve the therapeutic outcomes [33].

All together our results demonstrate that inhibition of FGFR1 signaling in SQCLC cell lines may impact on cancer cell growth also by affecting glucose energy metabolism. The AKT/mTOR pathway plays a key role in this regard, providing a rational for combining NVP-BGJ398 with PI3K/mTOR inhibitors in those cell models in which FGFR1 inhibition fails to down-regulate this signaling cascade. This conclusion is supported by a recent study demonstrating that activation of AKT signaling is a mechanism of acquired resistance to BGJ398 in lung and bladder cancer cell lines carrying activating FGFR alterations, and that such resistance can be efficaciously overcome by treatment with the AKT inhibitor GSK2141795 or by AKT silencing [34]. Interestingly, in our study not only the dual PI3K/mTOR inhibitor NVP-BEZ235 but also the selective inhibitor RAD001 improved the anti-tumor efficacy of NVP-BGJ398 treatment in both H1703 and LENTI-4 cells, underlining the relevance of mTOR inhibition for SQCLC. In LENTI-4 cells and tumor xenografts the combination of NVP-BGJ398 with NVP-BEZ235 was associated with a significant inhibition of src and FAK, one of its relevant downstream target. The importance of such signaling cascade in NSCLC tumorigenesis has been previously evidenced [35]. Notably, FAK, in addition to its canonical function as a modulator of integrin signaling, has been recently involved in the reprogramming of energy metabolism towards glycolysis [36]. Therefore, downregulation of src/FAK pathway, together with NVP-BEZ235-induced inhibition of the AKT/mTOR pathway, might contribute to the greater efficacy of the drug combination over individual treatments both *in vitro* and *in vivo*. Finally, the anti-tumor potency associated with down-regulation of GLUT-1 by FGFR1 and AKT/mTOR co-targeted inhibition was confirmed also *in vivo*, further strengthening the contention that tackling on cancer glucose metabolism may offer a new therapeutic option for the treatment of SQCLC.

## MATERIALS AND METHODS

### Cell culture

The human NSCLC cell lines H1703, H520, H596, SKMES-1, Calu-1 and H1581 were purchased from the American Type Culture Collection (ATCC, Manassas, VA); ATCC authenticates the phenotypes of these cell lines on a regular basis. All cells were cultured as recommended and maintained at 37°C in a humidified atmosphere of 5% CO_2_ and 95% air. Hypoxic conditions were established by placing the cells in a tissue culture incubator with controlled O_2_ levels (Binder GmbH, Tuttlingen, Germany).

### Drug treatment

Dovitinib (TKI258), NVP-BGJ398, NVP-BEZ235, and RAD001 were provided by Novartis International AG (Basel, Switzerland), PD173074 and imatinib mesylate were purchased from Selleckchem (Munich, Germany). U0126 was from Sigma-Aldrich (Saint Louis, MO). DASA was from Merck Millipore (Billerica, MA). Drugs were dissolved in DMSO (Sigma-Aldrich) and diluted in fresh medium before use. The final concentration of DMSO in medium never exceeded 0.1% (v/v) and equal amounts of the solvent were added to control cells. For the experiments of FGFR1 stimulation, the cells were incubated in Fetal Calf Serum-free (-FCS) RPMI 1640 medium added with sodium selenite (5 ng/ml) and BSA (0.25%). After 24h, the medium was replaced with fresh serum-free medium, the cells were pre-treated with or without drugs for 1h, and then stimulated with 25 ng/ml human FGF2 (Miltenyi Biotec, Bergisch Gladbach, Germany) for variable periods of time.

### Analysis of cell proliferation, cell death, and cell cycle

Cell proliferation was evaluated by counting the cells in a Bürker hemocytometer with trypan blue exclusion method and by Crystal Violet (CV) staining as previously described [37]. Cell death was assessed on cells stained with Hoechst 33342 and Propidium iodide (PI) using fluorescence microscopy as previously described [38]. Distribution of the cells in the cell cycle was determined by PI staining and flow cytometry as described elsewhere [38].

### Western blotting analysis

Procedures for protein extraction, solubilization, and protein analysis by 1-D PAGE are described elsewhere [22]. Antibodies against p-FGFR^Tyr653/654^, FGFR1, p-FRS2-α^Tyr196^, p-ERK1/2^Thr202/Tyr204^, ERK1/2, p-mTOR^Ser2448^, mTOR, p-AKT^ser473^, AKT, p-P70S6K^Thr389^, P70S6K, p-AMPKα1^Thr172^, p-src^Tyr416^, src, p-FAK^Tyr397^, FAK were from Cell Signaling Technology (Beverly, MA); the antibodies against GLUT-1 and AMPKα1 were from AbCam (Cambridge, MA); the antibody against HIF-1α was from BD Biosciences (Franklin Lakes, NJ); the antibody against actin was from Sigma-Aldrich. HRP-conjugated secondary antibodies were from Pierce (Rockford, IL) and chemiluminescence system (ImmobilionTM Western Chemiluminescent HRP Substrate) was from Millipore (Temecula, CA).

### Cell migration

The migration assay was carried out using a Transwell chamber with 6.5-mm-diameter polycarbonate filters (8-μm pore size) (BD Biosciences) as previously described [39].

### Spheroid generation

Spheroids were generated using LIPIDURE^®^-COAT PLATE A-U96 (NOF Corporation, Tokyo, Japan) as previously described [40]. Briefly, 500 cells were seeded in medium with 0.2% FCS and after 2 days (T0) the spheroids were treated with 1μM NVP-BGJ398 in the presence of FGF2 for further 7 days. The effect of the drug was evaluated in term of volume changes using the Nikon Eclipse E400 Microscope with digital Net camera. The volume of spheroids was measured [D=(Dmax+Dmin)/2; V=4/3π(D/2)3] with Image J software and the Fold Increase (FI) index was calculated as the ratio between the spheroid volume after 7 days and the volume at T0.

### Quantitative real-time PCR

Total RNA was isolated using RNeasy Mini Kit (Qiagen, Venlo, Netherlands). RNA (2 μg) was retrotranscribed using the High Capacity RNA-to-cDNA Kit (Applied Biosystems, Foster City, CA), according to the manufacturer's instructions. Primers to specifically amplify FGFR1 (Hs_FGFR1_1_SG [cat. no. QT00102837]) and GLUT-1 (Hs_SLC2A1_1_SG [cat. no. QT00068957]) were obtained from Qiagen. The quantitative real-time PCR was performed in a 20μL reaction volume containing Fast SYBR Green Master Mix (Applied Biosystems). All reactions were performed in triplicate using the StepOne system instrument (Applied Biosystems) as previously described [41]. Amplifications were normalized to HPRT1 (Hs_HPRT1_1_SG [cat. no. QT00059066]) and PGK1 (Hs_PGK1_1_SG [cat. no. QT00013776]). The fold change was calculated by the ΔΔCT method.

### RNA interference assay

Cells were transfected with Silencer^®^ Selected Validated siRNA (Ambion, Thermo Fisher, Waltham, MA) against FGFR1 (mixture of s5164, s5165, s5166) with a final concentration of 60 nM. Negative control (medium GC content and low GC content) was from Invitrogen, Thermo Fisher. The transfection was carried out as previously described [42].

### Generation of FGFR1 over-expressing cells by a lentiviral vector system

The lentiviral transfer vector (pTOL-Bsd) containing the FGFR1 sequence (DQ894999) was purchased from TransOmic (Huntsville, AL). According to manufacturer's instructions, SKMES-1 cells were seeded at the density of 7×10^4^ cells in a 24 well-plate with complete medium. Starting from 1.8×10^7^ TU/ml of viral particles, serial dilutions were prepared in serum-free medium containing 8μg/ml polybrene (Sigma-Aldrich) and added to each well. After an overnight-incubation, the viral supernatant was replaced with complete medium and the cells were incubated for further 24h. Then blasticidin 10μg/ml (Thermo Fisher) was added to the cultures for the selection of resistant cells. FGFR1 expression was then evaluated by both RT-PCR and Western Blot analysis.

### Glucose uptake

Glucose uptake was measured as described previously [22]. Briefly, cells were rinsed with Kreb's Ringer HEPES buffer (KRH) and incubated in KRH containing 2μCi/ml Deoxy-D-glucose-2-[1,2-^3^H(N)] (2DG, PerkinElmer, Waltham, MA) at 37°C for 5min. Then, the cells were quickly rinsed three times in fresh cold Earle's solution containing 0.1mM phloretin (Sigma-Aldrich). Ice-cold trichloroacetic acid (TCA, 5%) was added and radioactivity in the acid extracts was measured by liquid-scintillation in three or four independent determinations. Cell proteins, precipitated by TCA, were dissolved in 0.5N NaOH and their concentration determined by a dye-fixation method (Bio-Rad, Hercules, CA). Glucose uptake was calculated as pmol of 2DG/mg protein/5 min and expressed as percent vs control condition.

### Glycolysis

Glycolytic flux was measured as described previously [22]. Briefly, cells were washed in PBS, pre-incubated in drug-containing or drug-free KRH buffer for 30min, and then incubated with 10μCi/ml D-glucose[5-^3^H(N)] (PerkinElmer) and unlabeled D-glucose (10mM final concentration). After 1h at 37°C, the reaction was stopped by adding HCl (0.04M final concentration). ^3^H_2_O was separated from [5-^3^H]glucose by diffusion in an airtight container for 72h. Radioactivity was measured by liquid scintillation. Cell proteins, precipitated by HCl, were dissolved in 0.5N NaOH and their concentration determined by a dye-fixation method. Glucose utilization was calculated as described by Ashcroft et al. [43] and expressed as percent vs control condition.

### Measurements of ATP intracellular levels and lactate production

Cellular ATP changes were determined by a luminescence assay (ATPLite-1step, PerkinElmer). At the end of the experiments, media were collected and centrifuged to eliminate cell debris. Lactate in the media was determined spectrophotometrically by an enzymatic assay (Sigma-Aldrich) according to manufacturer's instructions. Lactate concentration was calculated as μmol/mg protein and expressed as percent vs control condition.

### PKM2 activity

The activity of PKM2 enzyme was measured on cell protein extracts using Pyruvate Kinase Activity Colorimetric Assay Kit (Biovision, Milpitas, CA) according to manufacturer's instructions. Absorbance was read at 570 nm. PKM2 activity was calculated as nmol/min/mg protein and data were expressed as percent vs control condition.

### *In vivo* study

A total of 5×10^6^ LENTI-4 cells were suspended in 200μL of Matrigel (BD Biosciences) and PBS (1:1) and were subcutaneously injected into both flanks of Balb/c-Nude female mice (Charles River Laboratories, Calco, Italy). The animals were housed in a protected unit for immunodeficient animals with 12-hour light-dark cycles and provided with sterilized food and water ad libitum. When tumor volume reached an average size of 200mm^3^ the animals were randomized into four groups (n=4 per group): control, NVP-BGJ398 (30 mg/Kg in 33% PEG300, 5% glucose), NVP-BEZ235 (10% N-Methyl-2-pyrrolidone, NMP, 90% PEG300) and the combined treatment. Drugs were given once per day five times per week by oral gavage. Control mice received vehicle according to the same schedules. Tumor xenografts were measured as previously described [41]. After 21 days of treatment, mice were humanely killed by cervical dislocation and tumors collected for further analyses. All experiments involving animals and their care were performed with the approval of the Local Ethical Committee of the University of Parma and by the Italian Ministry of Health in July 2013 (Protocol No. 50/13, 8 July 2013), in accordance with the institutional guidelines that are in compliance with national (DL116/92) and international (86/609/CEE) laws and policies.

### Morphometric analysis of tumor xenografts

The volume fraction of intact and necrotic neoplastic tissue and fibrosis was calculated on Masson's Trichrome and hematoxylin and eosin (H&E)–stained sections by microscopic examination at 200X magnification of adjacent fields to cover the entire area of each nodule. This analysis was performed with the aid of a grid defining a tissue area of 0.23mm^2^ and containing 42 sampling points each covering an area of 0.0052mm^2^. The number of points overlying each tissue components was counted and expressed as the percentage of the total number of points explored. Combining the entire tumor volume with the above morphometric measurements, the total volume occupied, respectively, by neoplastic, connective and necrotic tissue was computed on each sample.

### Immunohistochemical analysis of tumor xenografts

To document changes at tissue level in the expression of GLUT-1, sections of tumors from all experimental groups were exposed to immunofluorescent labeling using mouse monoclonal anti–GLUT-1 antibody (AbCam) followed by fluorescein isothiocyanate–conjugated secondary antibody (Sigma-Aldrich). Neoplastic expression of GLUT-1 was ascertained by the simultaneous detection of CD44. Briefly, GLUT-1-stained sections were incubated with rat monoclonal anti-CD44 antibody (HCAM-IM7, Santa Cruz Biotechnology, Dallas, TX) followed by specific Cy3-conjugated secondary antibody (1:50 Jackson ImmunoResearch Laboratories, West Grove, PA). Nuclei were counterstained with 4,6-diamino-2-phenylindole (DAPI). On sections from each experimental group, images of representative microscopic fields covering a tissue area of a minimum of 9.6mm^2^ to a maximum of 12.4mm^2^ were acquired with precalibrated gain and exposure time at 100x magnification using a fluorescence microscope (Olympus BX60).

The fractional area occupied by GLUT-1^pos^/CD44^pos^ cells was evaluated by computing fluorescent signals using a software for image analysis (Image Pro Plus 4.0, Media Cybernetics, Inc., USA).

### Statistical analysis

Statistical analyses were carried out using Graph-Pad Prism version 6.0 software (GraphPad Software, San Diego, CA). Statistical significance of differences among data was estimated by Student's t test or by one-way analysis of variance (ANOVA) followed by Bonferroni's post-test, and p values are indicated where appropriate in the figures and in their legends. p values less than 0.05 were considered significant. For the *in vivo* studies, comparison among groups was made using two-way repeated measures ANOVA followed by Bonferroni's post-test (to adjust for multiple comparisons). Adjusted p values less than 0.05 were considered significant.

## SUPPLEMENTARY MATERIALS FIGURES


