# Correction: Enhancement of the anti-tumor activity of FGFR1 inhibition in squamous cell lung cancer by targeting downstream signaling involved in glucose metabolism

**DOI:** 10.18632/oncotarget.28844

**Published:** 2026-03-27

**Authors:** Claudia Fumarola, Daniele Cretella, Silvia La Monica, Mara A. Bonelli, Roberta Alfieri, Cristina Caffarra, Federico Quaini, Denise Madeddu, Angela Falco, Andrea Cavazzoni, Graziana Digiacomo, Giulia Mazzaschi, Valentina Vivo, Elisabetta Barocelli, Marcello Tiseo, Pier Giorgio Petronini, Andrea Ardizzoni

**Affiliations:** ^1^Department of Medicine and Surgery, University of Parma, Parma, Italy; ^2^Food and Drug Department, University of Parma, Parma, Italy; ^3^Medical Oncology Unit, University Hospital of Parma, Parma, Italy; ^4^Division of Medical Oncology, Sant’Orsola-Malpighi University Hospital, Bologna, Italy; ^*^Joint last authors

**This article has been corrected:** In Figure 6D, a splice was identified in the GLUT-1 blot. The authors explained that this occurred while combining two blots at different exposures of the same experiment. To address this, the authors provided the original uncropped blot for GLUT-1, which was used to generate the correction.

The corrected Figure 6D is shown below. The authors declare that these corrections do not affect the results or conclusions of the paper.

Original article: Oncotarget. 2017; 8:91841–91859. 91841-91859. https://doi.org/10.18632/oncotarget.19279

**Figure 6:**
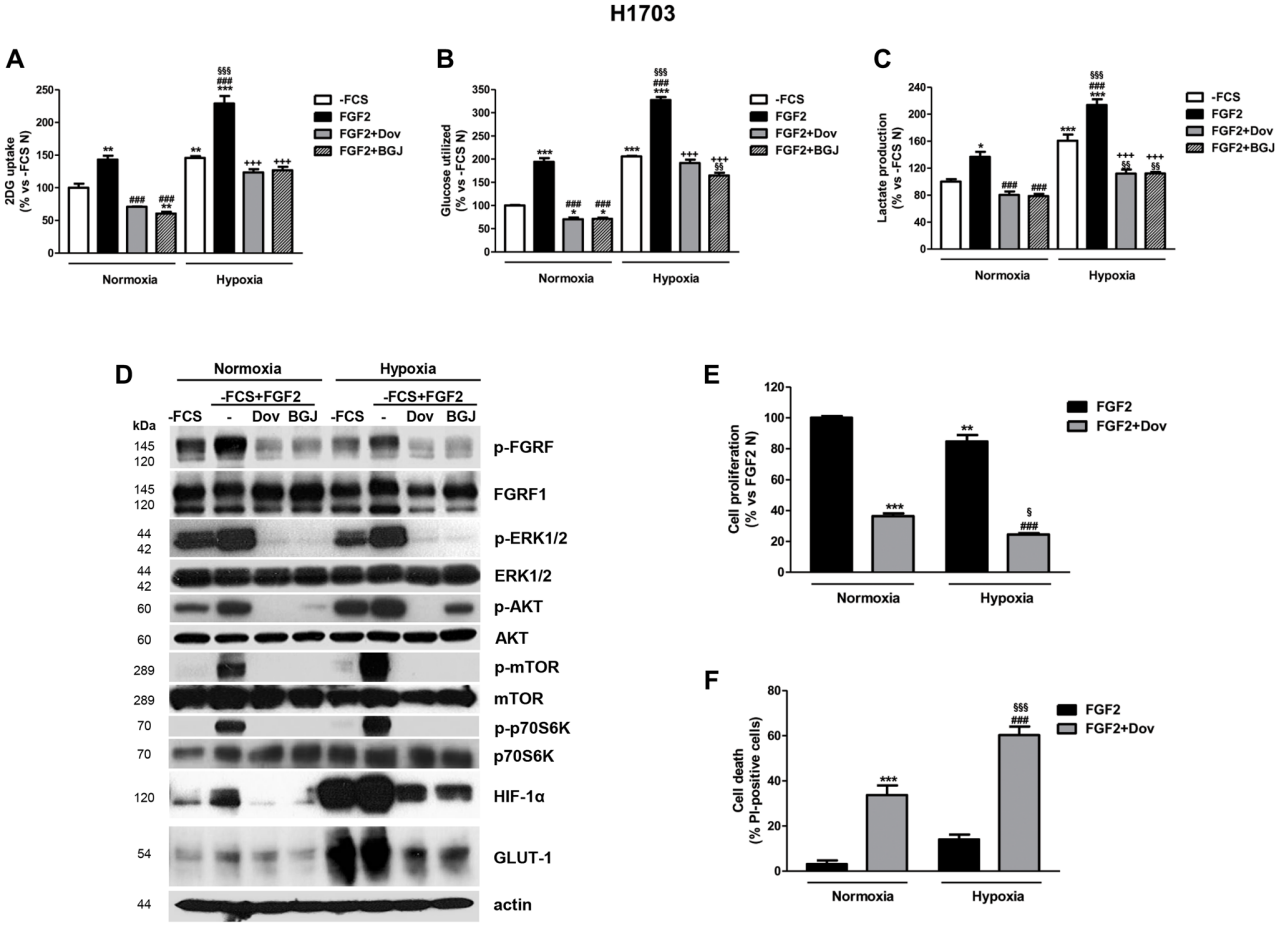
Effects of FGFR1 inhibition under FGF2 stimulation in serum-deprived H1703 cells in normoxic and hypoxic conditions. H1703 cells, cultured in -FCS for 24 h, were pre-incubated for 1 h with 1 μM dovitinib or NVP-BGJ398, stimulated with FGF2, and then incubated in normoxic and hypoxic (0.5% O_2_) conditions. After 16 h glucose uptake (**A**), glycolysis (**B**), and lactate production (**C**) were measured and protein expression from cell lysates was assessed by Western Blot analysis (**D**). After 48 h cell proliferation (**E**) and cell death (**F**) were evaluated by cell counting with trypan blue dye exclusion method and by fluorescence microscopy on Hoechst 33342/PI stained cells, respectively. Results are mean values ±SD of three independent determinations. Data in (a, b, and c) are expressed as percent versus -FCS control cells in normoxia (-FCS N). ^*^P < 0.05, ^**^P < 0.01, ^***^P < 0.001 vs. -FCS N; ^###^*P* < 0.001 vs. FGF2 N, ^§§^*P* < 0.01, ^§§§^*P* < 0.001 vs. -FCS Hypoxia (-FCS H), ^+++^*P* < 0.001 vs. FGF2 H. Data in (E) are expressed as percent versus FGF2 N. ^**^P < 0.01, ^***^P < 0.001 vs. FGF2 N; ^###^*P* < 0.001 vs. FGF2 H; ^§^*P* < 0.05, ^§§§^*P* < 0.001 vs. FGF2+Dovitinib N.

