# Supplementary material for: Enhancement of the anti-tumor activity of FGFR1 inhibition in squamous cell lung cancer by targeting downstream signaling involved in glucose metabolism
Source: Oncotarget. 2017 Jul 17;8(54):91841–59. doi: 10.18632/oncotarget.19279 (PMC5696146; doi:10.18632/oncotarget.19279)
Supplement: Supplementary file 1 [file oncotarget-08-91841-s001.pdf]

Enhancement of the anti-tumor activity of FGFR1 inhibition in squamous cell lung cancer by targeting downstream signaling involved in glucose metabolism

SUPPLEMENTARY MATERIALS

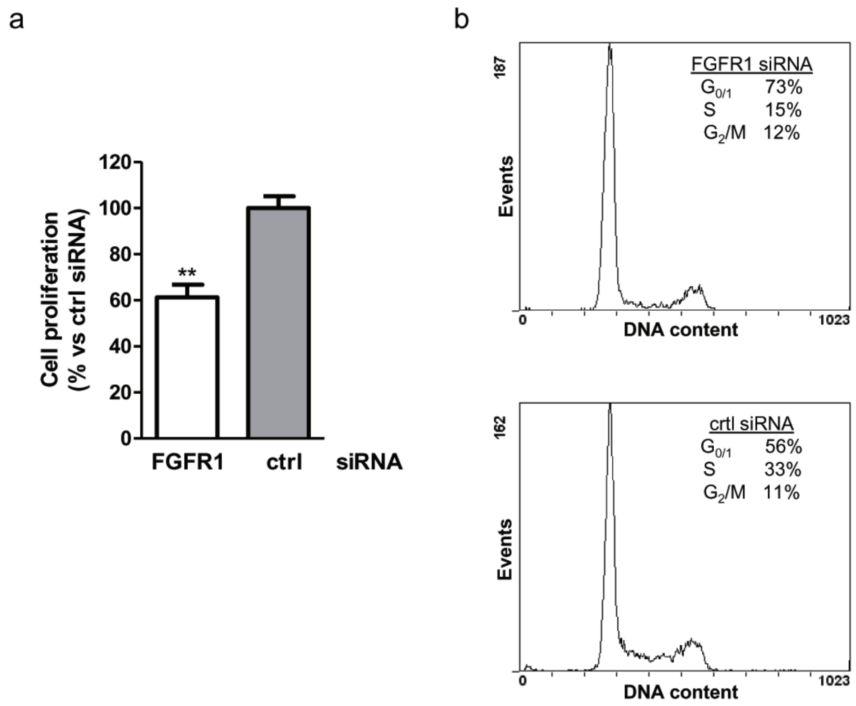

Supplementary Figure 1: Effects of FGFR1 silencing on cell proliferation and cell cycle phase distribution in H520 cells. H520 cells were treated with a pool of FGFR1 siRNA or control siRNA. **(a)** After 72h cell proliferation was assessed by cell counting with trypan blue exclusion method. Data are mean values ±SD of three independent experiments and are expressed as percent versus control siRNA. **P<0.01 vs control siRNA. **(b)** After 72h the cells were analyzed by flow cytometry for cell cycle phase distribution. Cytofluorimetric profiles and percentages of cells residing in each cycle phase are shown and are representative of two independent experiments.

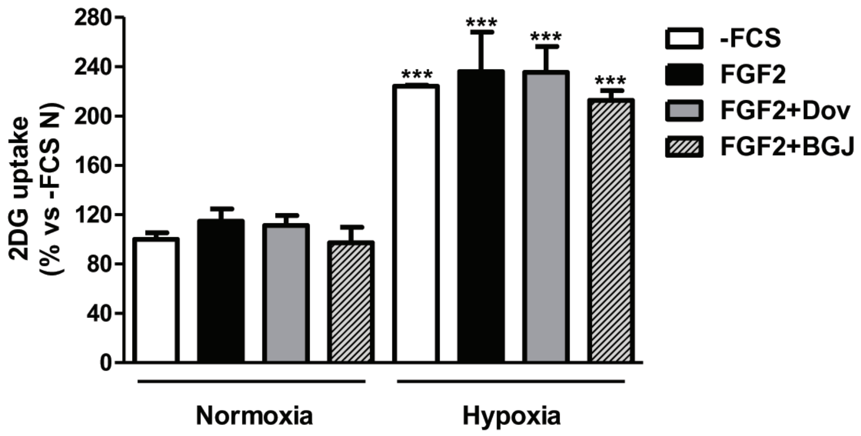

Supplementary Figure 2: Effects of FGF2 stimulation on SKMES-1 cells under normoxic and hypoxic conditions. SKMES-1 cells, cultured in -FCS for 24h, were pre-incubated for 1h with 1μM dovitinib or NVP-BGJ398, stimulated with FGF2, and then incubated in normoxic and hypoxic (0.5% O_2) conditions. Glucose uptake was measured after 16h. Data are expressed as percent versus -FCS control cells in normoxia (-FCS N) and are mean values ±SD of three independent experiments. ***P<0.001 vs -FCS N.
